# Generation of a Bioengineered Substitute of the Human Sclero-Corneal Limbus Using a Novel Decellularization Method

**DOI:** 10.3390/pharmaceutics17121540

**Published:** 2025-11-29

**Authors:** Paula Ávila-Fernández, David Sánchez-Porras, Miguel Etayo-Escanilla, Carmen González-Gallardo, Miguel Alaminos, Jesús Chato-Astrain, Fernando Campos, Óscar Darío García-García

**Affiliations:** 1 Tissue Engineering Group, Department of Histology, Faculty of Medicine, University of Granada, 18016 Granada, Spain; pavila@ugr.es (P.Á.-F.); davidsp@ugr.es (D.S.-P.); metayo@ugr.es (M.E.-E.); mariac.gonzalez.gallardo.sspa@juntadeandalucia.es (C.G.-G.); malaminos@ugr.es (M.A.); ogarcia@ugr.es (Ó.D.G.-G.); 2 Instituto de Investigación Biosanitaria ibs.GRANADA, 18012 Granada, Spain; 3 Doctoral Program in Biomedicine, University of Granada, 18016 Granada, Spain; 4 Division of Ophthalmology, Hospital Clínico Univeritario San Cecilio, 18007 Granada, Spain

**Keywords:** tissue engineering, sclero-corneal limbus, decellularization, advanced therapies, biofabrication

## Abstract

**Background**: Severe dysfunction of the human limbus associated with limbal stem cell deficiency is a therapeutic challenge, especially when a structural alteration of the limbal niche is associated. **Methods:** We have evaluated seven decellularization protocols applied to 20 human sclero-corneal limbus, based on the use of SDS (protocol P1), SDS + NaCl (P2), SDS + triton X-100 + SDC + enzymatic treatment (P3), SDS + triton X-100 + SDC + enzymatic treatment + trypsin (P4), sulfobetains + DNAse (P5), sulfobetains + SDC + DNAse (P6) and SDC + DNAse (P7). The decellularization efficiency of each protocol, biocompatibility and safety, as well as their capability to support cell attachment and differentiation, were evaluated. **Results:** Results showed that the use of protocols P1 to P4, based on strong ionic detergents such as SDS, was not efficient for decellularizing the human limbus. Conversely, protocols P5, P6 and P7 removed more than 95% of DNA while preserving 60–100% of the extracellular matrix components. These protocols were biocompatible, as macrophages cultured with decellularized scaffolds were viable and differentiated to the pro-regenerative M2 phenotype (CD163/CD86 ratio > 2) without inducing a significant increase in reactive oxygen species (ROS). Protocols P6 and P7 supported cell attachment, survival and differentiation of corneal epithelial cells and four types of mesenchymal stem cells cultured on the surface of these scaffolds. Cellularized limbi showed positive expression of several limbal cell markers, especially in scaffolds decellularized with protocol P6. **Conclusions:** These results support the use of protocol P6 for the generation of human limbal substitutes by tissue engineering using decellularized human limbi. Future studies should determine the clinical potential of the regenerative biomaterial generated in patients with structural limbal damage, particularly in patients with chemical burns and aniridia, where conventional stem cell therapies fail.

## 1. Introduction

The sclerocorneal limbus, a region allocated at the junction between the cornea and the sclera, has been identified as a niche of limbal epithelial stem cells (LESC) since 1970 [1]. Specifically, LESC reside at the basal layer of the palisades of Vogt, which are undulated structures of the human limbus in which the stroma and the epithelium are interconnected by projections of one tissue to the other one [2]. LESC function is critical for corneal maintenance and regeneration [3], but it can be disrupted by numerous conditions, including hereditary diseases, such as aniridia or dyskeratosis congenita, or acquired conditions, such as chemical and thermal injuries, multiple surgeries, or Stevens-Johnson and other autoimmune diseases [2,3,4,5]. It is well known that Limbal Stem Cell Deficiency (LSCD), whose incidence is estimated at 3.6 per 100,000 people [6], is typically associated with cornea conjunctivalization, vascularization, and opacification [2,4], leading to significant vision alterations, pain, photophobia, corneal erosion, vision loss, and blindness [2,3,4]. In recent years, the importance of the limbal microenvironment, or limbal niche, has been recognized in supporting LESC homeostasis and function [7]. This niche favors the normal interaction between diverse cell types, signaling factors, and the extracellular matrix (ECM), which are necessary for a normal functionality of the LESC residing in the niche [8,9]. Disruption of this complex structure can contribute to the development of LSCD [7].

Several approaches are currently available for the treatment of patients with LSCD, ranging from anti-inflammatory drugs for mild diseases to transplant surgery for severe LSCD [2]. In patients affected by unilateral LSCD, an autologous limbal transplant may be a therapeutic approach, although it can irreversibly damage the healthy limbus in the donor eye [2,4,5]. In bilateral cases, the limbal transplant graft must be obtained from a cadaveric or a live related donor, which requires prolonged immunosuppressive treatment, and results are typically suboptimal [5]. The use of LESC cultured and expanded in the laboratory as an advanced therapy medicinal product (ATMP) offers an interesting alternative to these patients [10]. However, this treatment is not able to restore the original structure of the limbal niche, and cases associated with severe structural damage of the limbus, which is common in chemical injuries and autoimmune diseases, cannot be treated with these novel therapies [11]. For these reasons, the development of novel therapeutic alternatives capable of restoring the structure and function of the human limbus is needed.

A promising solution for this problem is the generation of a bioartificial limbal substitute by tissue engineering [12]. Although several biofabrication methods can be used to generate a bioartificial tissue, the complex three-dimensional structure of the limbus is very difficult to reproduce in the laboratory [13]. In this regard, decellularization techniques have been proposed as efficient biofabrication methods allowing the generation of cell-free tissues and organs capable of preserving the native architecture and the ECM components of the native structures. Decellularized tissues are appropriate candidates for use as scaffolds, as they can support cell culture, while maintaining the natural microenvironment of the original organ [14]. Regarding the limbus, very few decellularization protocols have been described to date, although some of them have offered promising results. Most of these protocols require long decellularization times, typically ranging from 3 to 7 days in most protocols using detergents, resulting in a partial alteration of the native ECM [13,15,16,17], reinforcing the need to optimize these protocols. Among the previously described protocols, a group of them is based on the use of sodium dodecyl sulphate (SDS), which showed good decellularization efficiency and proper ECM preservation when applied to the porcine limbus [13,15]. Another group of protocols made use of sodium deoxycholate (SDC) and nuclease enzymes with promising outcomes and shorter decellularization times, resulting in an increased ECM preservation [16,17]. However, optimization of these methods to obtain a fully biomimetic decellularized limbal structure is still needed, and these protocols might not be fully functional on the human scleral limbus. To address these limitations, we developed two novel protocols (P5 and P6) combining sulfobetaine detergents with enzymatic treatment, reducing processing time by 60% while improving ECM preservation.

The objective of this study is to develop a human limbus substitute by using optimized decellularization protocols. To achieve this, we evaluated seven decellularization protocols, including five previously described protocols and two novel protocols developed by the research group (P5 and P6). Additionally, the potential of each decellularized human limbus to support cell attachment and differentiation was analyzed.

## 2. Materials and Methods

### 2.1. Generation of Human Decellularized Limbal Substitutes

A total of 20 human sclerocorneal rings were obtained as remaining material that is normally discarded after the penetrating keratoplasty surgery performed in patients with severe corneal damage. All donors were cadaveric, with an average donor age of 59 years. On arrival to the laboratory, rings were washed in Dulbecco’s phosphate-buffered saline-PBS- (Merck, Darmstadt, Germany) and frozen at −80 °C using a cryopreservation solution consisting of 10% dimethyl-sulfoxide (Panreac AppliChem, Barcelona, Spain) in fetal bovine serum-FBS- (Merck, Darmstadt, Germany) until the moment of use. Limbi were then thawed, washed in PBS supplemented with 1% antibiotic/antimycotic solution (Merck, Darmstadt, Germany), and any remnants of conjunctiva, iris, or other tissues that may be attached to the limbus were carefully removed. Rings were then sectioned into six identical fragments of approximately 4 mm in length, as previously described [13].

Once obtained, the limbal ring fragments were subjected to the seven decellularization protocols (P1 to P7) that are summarized in Table 1. Protocols P1, P2, P3, P4 and P7 were previously described in the literature, whereas P5 and P6 were developed ad hoc for the present work. In brief, the different protocols were carried out as follows:

- Protocol 1 (P1) [13]: Double-distilled water (ddH_2_O) for 24 h; 0.1% SDS (3 incubations of 24 h each) (Sigma-Aldrich, St Louis, MO, USA); PBS (5 incubations of 15 min each).
- Protocol 2 (P2) [13]: ddH_2_O for 24 h; 0.1% SDS for 24 h; 1.5 M of sodium chloride (NaCl) (Merck, Darmstadt, Germany); PBS (5 incubations of 15 min each).
- Protocol 3 (P3) [13]: ddH_2_O for 24 h; 0.1% SDS for 24 h; 3 washes in ddH_2_O (30 min each); 0.6% of Triton X-100 (Sigma-Aldrich, St Louis, MO, USA) for 24 h; 3 washes in ddH_2_O (30 min each); 1% of SDC for 24 h; 3 washes in ddH_2_O (30 min each); 100 mg/L of DNAse (Sigma-Aldrich, St Louis, MO, USA) and 20 mg/L of RNAse (Sigma-Aldrich, St Louis, MO, USA) for 45 min; PBS (5 incubations of 15 min each).
- Protocol 4 (P4) [13]: ddH_2_O for 24 h; 0.1% SDS for 24 h; 3 washes in ddH_2_O (30 min each); 0.6% of Triton X-100 for 24 h; 3 washes in ddH_2_O (30 min each); 1% SDC (Sigma-Aldrich, St Louis, MO, USA) for 24 h; 3 washes in ddH_2_O (30 min each); 0.05% of Trypsin (Sigma-Aldrich, St Louis, MO, USA) for 1 h; 100 mg/L of DNAse and 20 mg/L od RNAse for 45 min; PBS (5 incubations of 15 min each).
- Protocol 5 (P5): 3 washes in ddH_2_O (15 min each); 0.6 mM of sulfobetaine 16 (SB-16) (Sigma-Aldrich, St Louis, MO, USA) and 125 mM of sulfobetaine 10 (SB-10) (Sigma-Aldrich, St Louis, MO, USA) for 1 h; 3 washes in PBS (30 min each); 1 mg/mL DNAse for 2 h; 4 washes in PBS (30 min each).
- Protocol 6 (P6): 3 washes in ddH_2_O (15 min each); 0.6 mM of SB-16 and 125 mM of SB-10 for 1 h; 3 washes in PBS (30 min each); 0.3% of SDC for 30 min; 3 washes in PBS (30 min each); 1 mg/mL DNAse for 2 h; 4 washes in PBS (30 min each).
- Protocol 7 (P7) [14]: 3 washes in ddH_2_O (15 min each); 1% of SDC for 30 min; 3 washes in PBS (30 min each); 1 mg/mL DNAse overnight (O.N.); 4 washes in PBS (30 min each).

### 2.2. Decellularization Efficiency Analysis

Efficiency of the decellularization protocols evaluated in this work was determined by quantifying the amount of DNA and cell remnants in the tissues subjected to each protocol. For DNA quantification, we first extracted total DNA from each sample using a QIAamp DNA Mini Kit (Qiagen, Hilden, Germany). In brief, decellularized fragments were dried and weighed and subsequently subjected to lysis following the kit manufacturer’s instructions. Isolated DNA quantification was performed using DNAds Quant-iT PicoGreen (Thermo Fisher, Waltham, MA, USA) according to the manufacturer’s recommendations and analyzed with an Infinite 200 Pro-MPlex fluorescence reader (Tecan, Männedorf, Switzerland) at 480/520 nm (Ex/Em). A standard curve was made using known concentrations of DNA, which allowed us to quantify the results. Results were then normalized to the dried weight of each sample and normalized versus the human native limbus that was not subjected to decellularization and was considered as a control (CTR) with 100% of DNA content. A total of six samples were analyzed per protocol.

Analysis of cell remnants was performed by staining any residual DNA in the decellularized tissues, as previously described [18]. For this, samples were fixed for 24 h in 4% formaldehyde, dehydrated and embedded in paraffin following routine histological methods. Then, 5 µm tissue sections were obtained using a microtome, mounted on glass slides, dewaxed and rehydrated for histological analysis. To identify cell nuclei and to evaluate the general structure of the tissues, sections were stained with hematoxylin-eosin (HE) following routine protocols. In brief, samples were incubated for 3 min in hematoxylin, differentiated in tap water and stained in eosin for 1 min (all these reagents, from Panreac AppliChem, Barcelona, Spain). Samples were coverslipped using mounting medium (Vector Laboratories, Newark, CA, USA), and images were obtained with a Pannoramic Flash Desk DW histological scanner (3DHISTECH, Budapest, Hungary). The use of this scanner ensures that all images are obtained under the same conditions and using the same background and white balance parameters. To specifically stain the DNA present in each sample, tissue sections were stained with 4’,6-diamidino-2-phenylindole (DAPI) (Vector Laboratories), coverslipped and analyzed using a Nikon Eclipse i90 fluorescent microscope(Tokyo, Japan) A total of three samples were analyzed per protocol.

### 2.3. ECM Preservation Analysis

To evaluate the structure and composition of the ECM, rehydrated tissue sections were subjected to different histochemical methods, including picrosirius red (PSR) for collagen staining, alcian blue pH 2.5 (AB) for proteoglycans, and periodic acid-Schiff (PAS) for glycoproteins, as previously described [19]. For PSR, slides were incubated in Sirius red F3B diluted in picric acid for 30 min and counterstained with Harris hematoxylin for 5 min. For AB, samples were incubated in 3% aqueous acetic acid solution for 3 min, followed by 30 min in AB working solution and stained with nuclear fast red for 1 min. Finally, PAS staining was carried out by incubating tissue sections in 0.5% periodic acid for 5 min, followed by incubation in Schiff reagent for 15 min and counterstaining with Harris hematoxylin for 1 min. All samples were coverslipped and scanned using a Pannoramic Flash Desk DW histological scanner. The histochemical signal obtained for each method was quantified using the ImageJ software (version 1.53 k, National Institute of Health, Bethesda, MD, USA) as described in previous studies [20]. Briefly, the positive staining intensity was quantified in a tissue area of 8300 µm^2^, with four measurements obtained per sample, and three samples per condition. Values were normalized to the CTR group, considered as 100% of the positive signal.

### 2.4. Ex Vivo Biocompatibility Evaluation

In order to evaluate the potential cytotoxic effects of tissues subjected to decellularization and their capability to drive a pro-inflammatory response, we carried out several ex vivo co-culture analyses using a macrophage cell line. These experiments were performed on the three decellularized limbi experimental groups that showed the most promising results in the analyses of decellularization efficiency and ECM preservation described above.

For these ex vivo studies, the macrophage cell line RAW.264, obtained from the American Type Culture Collection (ATCC, Manassas, VA, USA) was cultured in a culture medium consisting of high glucose Dulbecco’s modified Eagle’s medium -DMEM- supplemented with 10% FBS and 1% of a cell culture antibiotic/antimycotic solution (all of them, from Merck). Ex vivo assays were performed in indirect contact between cells and decellularized tissues using a co-culture system. First, macrophages were seeded in 24-well plates at a cell density of 60,000 cells/cm^2^. Once the cells were attached, Transwell porous inserts were placed on each well, and a decellularized limbal substitute was placed into each insert, immersed in culture medium to allow the molecules to flow from the tissue to the cells cultured below. Culture plates were kept for 24 h in a cell culture incubator using standard culture conditions (37 °C and 5% CO_2_). Two control groups were used: (1) a control group of non-activated macrophages (CTR), consisting of macrophages cultivated in their normal culture medium; (2) a control group of activated macrophages (aCTR), in which macrophages were activated with 1 µg/mL of bacterial endotoxin (lipopolysaccharide or LPS) (Merck) diluted in their culture medium.

#### 2.4.1. Assessment of the Potential Cytotoxic Effects of the Tissues Subjected to Decellularization

To evaluate the cytotoxic effects of the decellularized tissues, we first analyzed the percentage of dead cells in the co-culture system using a Live/Dead viability/cytotoxicity kit (Invitrogen/Thermo Fisher, Waltham, MA, USA), which allowed us to discern between viable and dead cells (green and red, respectively). In brief, cells previously co-cultured with the decellularized tissues were washed twice with PBS and incubated in the Live/Dead reagent solution for 15 min. Cells were then analyzed using a ZOE Fluorescent Cell Imager (Bio-Rad, Hercules, CA, USA) to determine the percentage of live and dead cells in each sample. These studies were carried out using 4 samples per condition (*n* = 4).

In addition, we analyzed the potential of the decellularized tissues to modify cell proliferation of co-cultured cells. With this purpose, the number of cells in an area of 0.16 mm^2^ was quantified in each culture at the end of the 24 h follow-up period. Results were expressed as the number of cells found per mm^2^ of culture surface (cell density). These studies were carried out using 4 samples per condition (*n* = 4).

Furthermore, the potential cytotoxic effects were evaluated by analyzing the oxidative stress response induced by each decellularized tissue on co-cultured macrophage cells, as determined by reactive oxygen species (ROS) factors quantification using a CellROX Deep Red Reagent (Invitrogen/Thermo Fisher, Waltham, MA, USA). In brief, cells were incubated for 30 min with the CellROX reagent at a final concentration of 5 µM, and the fluorescent reaction was assessed using an Infinite 200 Pro-MPlex microplate reader at 644/680 nm (Ex/Em). As control, a group in which ROS factors were induced by the addition of 5 mM H_2_O_2_ for 15 min (R-CTR) was used, and all results were normalized to the number of cells in each plate. These studies were carried out using 4 samples per condition (*n* = 4).

#### 2.4.2. Evaluation of the Potential Pro-Inflammatory Effects of the Tissues Subjected to Decellularization

To determine the potential of decellularized tissues to drive a pro-inflammatory response, we assessed macrophage polarization as previously reported [21]. Briefly, macrophages were co-cultured with the different decellularized tissues for 24 h. Subsequently, cells were fixed for 10 min in 4% formaldehyde and washed twice with PBS. Then, cells were resuspended in a commercial casein solution (Vector Laboratories, Burlingame, CA, USA) and incubated for 15 min with CD86 antibodies (Invitrogen/Thermo Fisher, Waltham, MA, USA), as a marker of pro-inflammatory phenotype (M1), and CD163 antibodies (Biolegend, San Diego, CA, USA), as a marker of pro-regenerative phenotype (M2), at final concentrations of 0.125 µg/test and 0.1 µg/test, respectively. The percentage of M1 and M2 cells was determined in each sample, as well as the rate M2/M1, and CTR and aCTR cells were used as controls. A total of four samples per condition were analyzed (*n* = 4).

### 2.5. Generation of Cellularized Limbal Substitutes by Tissue Engineering

To generate in the laboratory a human limbus substitute by tissue engineering, we first selected the decellularized tissues showing the best results in the characterization analyses described above. Then, selected decellularized tissues were recellularized with 5 human cell types, including LESC and 4 different mesenchymal stem cells (MSC). Normal human LESC primary cultures were purchased to Innoprot (P10871, Derio, Spain) and cultured with epithelial cell culture medium containing a 2:1 mixture of DMEM and Ham F-12 (Merck) supplemented with 10% FBS, 1% antibiotic/antimycotic solution, 24 µg/mL adenine, 5 mg/mL human insulin, 10 ng/mL epidermal growth factor, 1.3 ng/mL triiodothyronine and 0.4 mg/mL hydrocortisone (all these components, from Merck). MSCs were isolated from small human tissue biopsies that were enzymatically digested and cultured using previously described protocols [22], and primary cell cultures were established of human adipose-derived stem cells (ADSCs), bone marrow-derived stem cells (BMSCs), dental pulp-derived stem cells (DPSCs) and umbilical cord Wharton’s Jelly-derived stem cells (WJSCs). ADSC, BMSC and DPSC were cultured in DMEM supplemented with 10% FBS and 1% antibiotic/antimycotic solution, whereas WJSC were cultured in DMEM Advanced culture medium (Thermo Fisher). All cell types were maintained in standard culture conditions (37 °C and 5% CO_2_).

To generate cellularized limbal substitutes by tissue engineering, cultured cells were trypsinized and subcultured on top of the selected limbal substitutes at a density of 150,000 cells per sample. To favor cell attachment, the decellularized tissues were temporarily placed on 2% type-I agarose molds and cells were allowed to attach to the scaffolds for 2 h before the tissues were covered with culture medium. Cellularized tissues were kept in culture for 7 days using the culture medium in which primary cell cultures were kept.

### 2.6. Histological, Immunohistochemical and Immunofluorescence Evaluation of the Cellularized Limbal Substitutes Generated by Tissue Engineering and Controls

Limbal substitutes and human native limbi used as CTR were fixed in 4% formaldehyde for 24 h, dehydrated using ethanol series and embedded in paraffin, following standard histological methods, and 5 µm tissue sections were obtained using a microtome. For histological analysis, sections were deparaffinized and rehydrated using decreasing concentrations of ethanol (Panreac AppliChem, Barcelona, Spain). For a general histological and morphological analysis of tissue structure and cell attachment and stratification, samples were stained with HE as described for decellularized tissues. To analyze cell viability in cellularized tissues, cells undergoing apoptosis were identified using a terminal deoxynucleotidyl transferase dUTP nick end labeling (TUNEL) assay (Promega, Madison, WI, USA), following the manufacturer’s instructions. In brief, tissue sections were incubated in a 20 μg/mL solution of proteinase K for 5 min, incubated in a TUNEL reaction solution for 1 h.

In addition, immunohistochemical and immunofluorescence analyses were performed to analyze cell proliferation and cell differentiation in the cellularized tissues. For cell proliferation, we quantified the percentage of cells showing a positive signal for the cell proliferation marker PCNA using immunohistochemistry. For epithelial cell differentiation, cells were evaluated for the expression of the corneal epithelium stem cell marker Δnp63, pancytokeratin AE1/AE3, cytokeratin 5 (KRT5), cytokeratin 12 (KRT12), cytokeratin 15 (KRT15), crystallin αA (CRYαA) and crystallin λ (CRY-λ). The technical details of the immunodetection procedures and antibodies used are summarized in Supplementary Table S1. For tissues stained with HE, or subjected to immunohistochemistry for AE1/AE3, KRT5, KRT12 and CRY-λ, samples were dehydrated and coverslipped using mounting medium (Vector Laboratories, Newark, CA, USA), and images were obtained with a Pannoramic Flash Desk DW histological scanner (3DHISTECH, Budapest, Hungary). For tissues subjected to immunofluorescence (TUNEL, PCNA, Δnp63, KRT15 and CRYαA), unstained cell nuclei were counterstained with a mounting medium containing DAPI, and slides were observed and photographed using a Nikon Eclipse i90 fluorescence microscope (Tokyo, Japan). Results of the immunohistochemistry and immunofluorescence analyses were semiquantitatively analyzed by three independent histologists to reduce potential biases using a previously reported scale [23], and the positive staining signal was rated as strong (+++), moderate (++), slight (+), very slight (±), or negative (−).

### 2.7. Statistical Analyses

The quantitative results obtained in the present study were analyzed using the Shapiro–Wilk normality test. Since not all quantitative distributions met the criteria for parametric analysis, statistical comparisons were then performed using the Kruskal–Wallis and Mann–Whitney nonparametric tests. A statistical *p* value < 0.05 was considered statistically significant for the two-tailed comparison tests. The statistical analysis was performed using the Real Statistics Resource Pack software (Release 7.2) (Dr. Charles Zaiontz, Purdue University (West Lafayette, IN, USA)), available at www.real-statistics.com (accessed on 20 October 2024).

## 3. Results

### 3.1. Evaluation of Decellularization Efficiency

**Figure 1:**
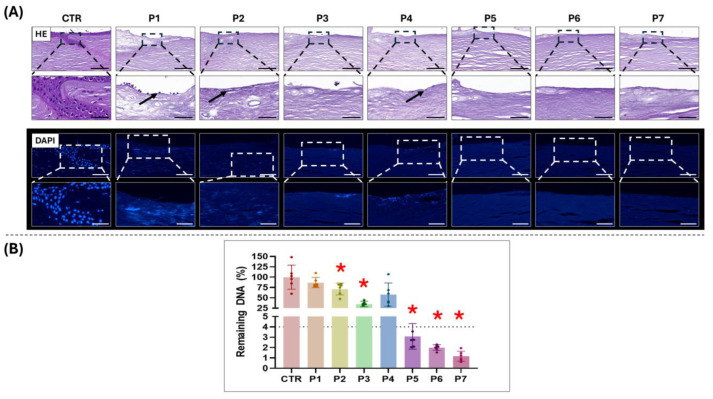
Assessment of decellularization efficiency in limbal substitutes subjected to seven decellularization protocols (P1 to P7) as compared to the native human limbus used as control (CTR). (**A**) Histological evaluation of CTR and decellularized limbal substitutes using hematoxylin-eosin (HE) and 4’,6-diamidino-2-phenylindole (DAPI) staining techniques. For each analysis technique, lower magnification images are shown on top, whilst higher magnification images below correspond to the inserts on the top images. Scale bars = 200 µm (HE lower magnification), 100 µm (DAPI lower magnification) and 50 µm (HE and DAPI at higher magnification). Black arrows indicate detectable residual cells. (**B**) Quantification of residual DNA content, normalized to the dry weight of the samples and expressed as percentage of residual DNA relative to the CTR, considered as 100% DNA content. Error bars represent standard deviations. The dotted line in Figure 1B corresponds to the threshold values recommended by the previous literature. Statistically significant differences to the CTR as determined with the Mann–Whitney test are labeled with asterisks (*).

To evaluate the decellularization efficiency of the different protocols used in the present study, we first analyzed the presence of residual cells and DNA in each type of sample. As shown in Figure 1A, histological evaluation of the decellularized tissues using HE and DAPI staining revealed a clear reduction in nuclear content with the use of all protocols, whereas the CTR native human limbus contained a high amount of cell nuclei. However, notable differences in decellularization efficiency were observed among the different protocols. Specifically, HE staining revealed the presence of detectable residual cells at the surface of limbal substitutes in P4 and, especially, in P1. In addition, we found a positive DAPI signal, suggesting the presence of cell nuclei or nuclear debris, in P1, P2, P3 and P4, with a higher positive signal in P1. In contrast, P5, P6 and P7 successfully removed all nuclei and nuclear debris as determined by both HE and DAPI staining.

Then, we extracted and quantified total DNA from each sample in order to determine the residual DNA remaining in each tissue subjected to decellularization. As shown in Figure 1B and Supplementary Table S2, results confirmed that the DNA content tended to decrease in all decellularized tissues. However, only P2, P3, P5, P6 and P7 showed a significant decrease compared to the native human limbus used as the CTR group, whereas P1 and P4 were not significantly different from CTR.

### 3.2. Analysis of ECM Preservation

**Figure 2:**
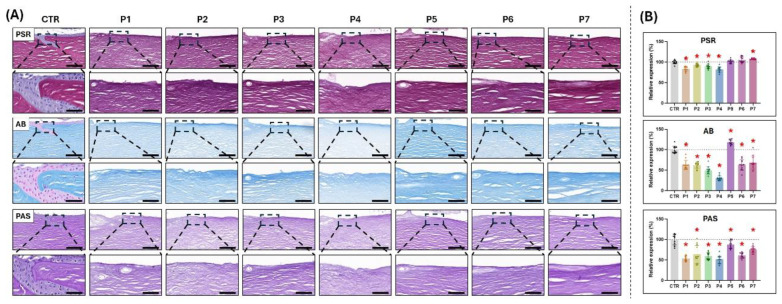
Evaluation of relevant extracellular matrix (ECM) components in limbal substitutes subjected to seven decellularization protocols (P1 to P7) as compared to the native human limbus used as control (CTR). (**A**) Histochemical analysis of CTR and decellularized limbal substitutes using picrosirius red (PSR), alcian blue (AB) and periodic acid-Schiff (PAS) staining methods. For each analysis technique, lower magnification images are shown on top, whilst higher magnification images below correspond to the inserts on the top images. Scale bars = 200 µm (lower magnification images) and 50 µm (higher magnification images). (**B**) Quantitative analysis of the PSR, AB and PAS staining intensity. Results were normalized with respect to the CTR, considered as 100% staining intensity. Error bars represent standard deviations. Statistically significant differences with the CTR group are labeled with asterisks (*).

In general, histochemical assessment of ECM preservation in tissues decellularized with the seven protocols described in this work resulted in a proper preservation of the general structure of the different tissues, although differences with CTR were detected in some groups of samples (Figure 2 and Supplementary Table S2). Specifically, when collagen fibers were analyzed using PSR staining, we found a significant signal intensity decrease in tissues decellularized with the P1, P2, P3 and P4 protocols, and an increase in P7, but the signal observed in P5 and P6 was not significantly different from CTR. Then, the analysis of the ECM proteoglycans as determined by AB staining demonstrated a significant decrease in the staining signal in the P1, P2, P3, P4, P6 and P7 groups, and a significant increase in P5. In turn, glycoproteins identified by PAS histochemistry revealed a significant decrease in these components in all samples, as compared to CTR, with the highest levels corresponding to the P5 and P7 protocols (Figure 2).

### 3.3. Ex Vivo Biocompatibility Analysis of Selected Decellularized Tissues

**Figure 3:**
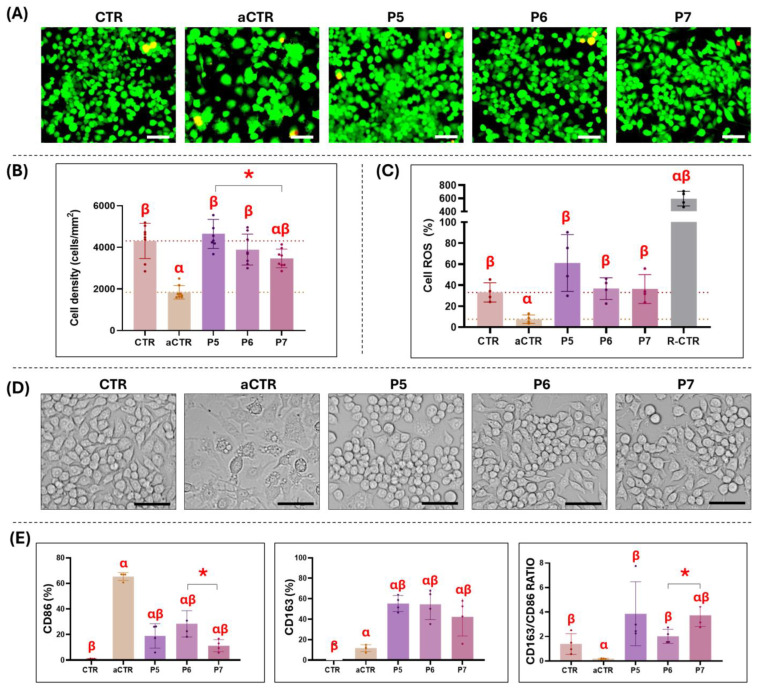
Ex vivo biocompatibility analysis of the human limbi decellularized with the selected protocols P5, P6 and P7, as compared with control cells. (**A**) Live/dead analysis of cell viability. Live cells are shown in green, whereas dead cells appear in red. (**B**) Cell density quantification after 24 h of co-culture with the different decellularized tissues and controls. (**C**) Analysis of reactive oxygen species (ROS factors) generated by cells co-cultured with the different decellularized tissues and controls, including a control of cells treated with H_2_O_2_ (R-CTR). (**D**) Brightfield images of cells co-cultured with the different decellularized tissues and controls to reveal cell morphology. Scale bar = 50 µm. (**E**) Assessment of macrophage polarization, represented as the percentage of CD86 and CD163 positive cells and the CD163/CD86 ratio. CTR: non-differentiated control cells cultured without any decellularized tissue; aCTR: control cells cultured without any decellularized tissue activated with LPS. Error bars represent standard deviations. Statistically significant differences with CTR cells are labeled with α, statistically significant differences with aCTR cells are labeled with β, whereas statistically significant differences between conditions are labeled with asterisks (*).

Once we selected protocols P5, P6 and P7 based on the results of the previous analyses, we analyzed the ex vivo biocompatibility of the human limbus decellularized with these three protocols. On the one hand, our analysis of cell viability of cells co-cultured with the decellularized limbi using Live/dead revealed that these tissues were not cytotoxic, and all cells showed high cell viability, with no differences versus CTR and aCTR cells (Figure 3A). On the other hand, evaluation of cell proliferation as determined by cell quantification showed non-significant differences in cell density when cells were cultured with substitutes decellularized with protocols P5 and P6. However, significant differences were found between CTR cells and cells cultured in the presence of scaffolds decellularized with protocol P7, as well as between CTR and aCTR cells (Figure 3B). In addition, we evaluated the cytotoxic effects of decellularized limbi by quantifying ROS activity in each study group. As shown in Figure 3C, co-culture of cells with scaffolds decellularized with protocols P6 and P7 resulted in the production of ROS factors that were very similar to CTR cells. However, co-culture with biomaterials corresponding to the P5 group resulted in an increase in ROS factors, although statistical significance was not reached.

To evaluate the potential pro-inflammatory effects of the decellularized tissues, we examined the expression of the specific markers CD86 and CD163 in cells co-cultured with the decellularized limbi. As displayed in Figure 3D,E, the CTR group of cells contained low amounts of CD163-positive cells and very low amounts of CD86-positive cells, resulting in a CD163/CD86 ratio of approximately 1.5, suggesting a M2 phenotype. These cells displayed a spherical, cobblestone morphology that was compatible with normal macrophage cells kept in culture. As expected, aCTR cells showed an important increment of CD86-positive cells, with a significant reduction in the CD163/CD86 ratio, suggesting an M1 phenotype. Interestingly, aCTR cells showed a marked morphological change, with flattened, star-shaped cells with numerous cell protrusions and intracellular granules and vesicles. In the case of cells co-cultured with the different decellularized scaffolds, we found a significant increase in the percentage of CD86-positive cells, although at a lower level than aCTR, and a very high increase in the percentage of CD163-positive cells, resulting in a CD163/CD86 ratio above 2 that was comparable or higher than the CTR group and significantly higher than the aCTR group. These results are compatible with an M2 phenotype in the P5, P6 and P7 groups.

### 3.4. Characterization of Cellularized Limbal Substitutes Generated by Tissue Engineering

**Figure 4:**
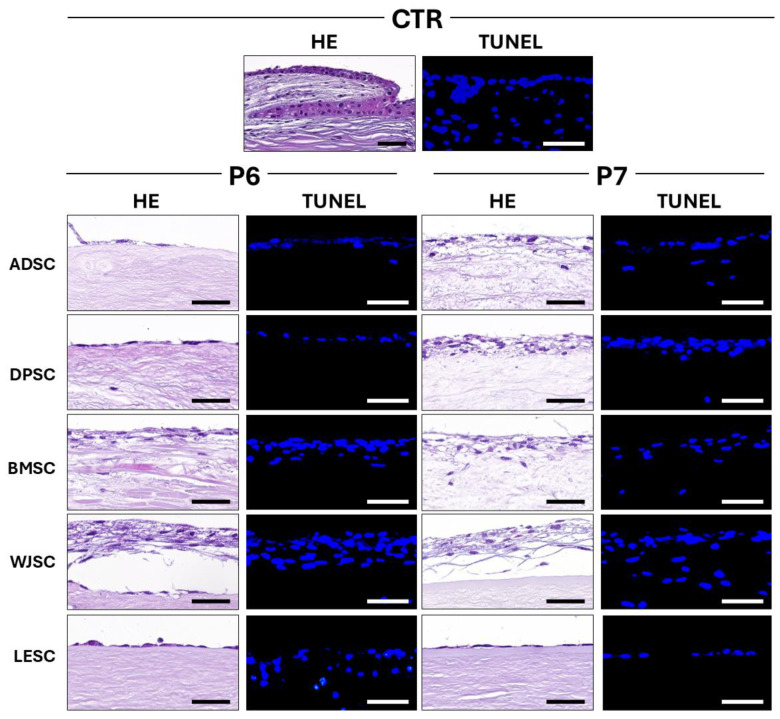
Evaluation of the cellularized human limbal substitutes generated by tissue engineering using decellularization protocols P6 and P7 and control tissues (CTR) to determine tissue structure and cell viability. HE: Hematoxylin-eosin staining used to evaluate the general structure of each tissue and to assess cell attachment and stratification; TUNEL: Terminal deoxynucleotidyl transferase dUTP nick end labeling used to identify apoptotic cells. ADSC: Limbal substitutes recellularized with human adipose-derived stem cells; DPSC: Limbal substitutes recellularized with dental pulp-derived stem cells; BMSC: Limbal substitutes recellularized with bone marrow-derived stem cells; WJSC: Limbal substitutes recellularized with Wharton’s Jelly-derived stem cells; LESC: Limbal epithelial stem cells. Scale bar = 50 µm.

Cellularization was carried out using LESC and four types of MSC on scaffolds decellularized with protocols P6 and P7, considered as the biomaterials showing the best results in the biocompatibility analyses described above. When the structure of each tissue substitute was evaluated using HE staining (Figure 4), we found that CTR limbi exhibited the typical structure of limbal cells, organized in Vogt palisades with several cell layers, whereas P6 and P7 limbal substitutes showed a proper cell attachment to the biomaterial surface, but were devoid of these specialized structures. Although cells tended to attach to the biomaterial in all groups, some structural differences were found when the epithelial layer was analyzed. As shown in Figure 4, the use of MSC was associated with a higher number of cell layers developed on the decellularized scaffold, especially when WJSC was used, followed by BMSC, whereas tissue substitutes generated with LESC contained only a single cell layer on the surface of the biomaterial. Interestingly, the structure of the epithelial layer was very similar in P6 and P7, with no relevant differences found between the two study groups.

**Figure 5:**
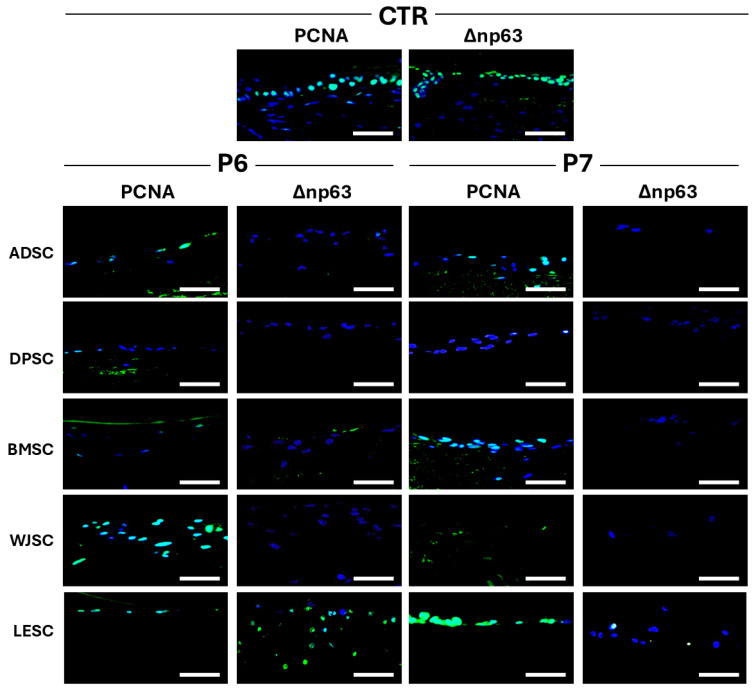
Evaluation of the cellularized human limbal substitutes generated by tissue engineering using decellularization protocols P6 and P7 and control tissues (CTR) to determine proliferation and limbal phenotype. PCNA: immunohistochemistry for the cell proliferation marker PCNA, revealing proliferating cells; Δnp63: immunohistochemical analysis of cells showing limbal phenotype as determined by the expression of the limbal marker Δnp63. ADSC: Limbal substitutes recellularized with human adipose-derived stem cells; DPSC: Limbal substitutes recellularized with dental pulp-derived stem cells; BMSC: Limbal substitutes recellularized with bone marrow-derived stem cells; WJSC: Limbal substitutes recellularized with Wharton’s Jelly-derived stem cells; LESC: Limbal epithelial stem cells. Scale bar = 50 µm.

Additionally, cell viability assays performed on the cellularized tissue substitutes using the TUNEL method revealed no apoptotic cells in any of the study groups, with results comparable to the CTR group, suggesting that cells in the cellularized limbal substitutes were viable. However, our analysis of cell proliferation found some differences related to the different cell sources used during the cellularization process. As shown in Figure 5 and Table 2, quantification of cells showing active proliferation determined by PCNA expression revealed that cells in the native CTR had high proliferation rates. In cellularized tissues, we found some differences between protocols P6 and P7. In cellularized scaffolds generated with protocol P6, PCNA expression was strong in WJSC samples, moderate in the LESC and ADSC groups and slight or very slight when DPSC or BMSC were used. In scaffolds decellularized with protocol P7, we found a moderate PCNA signal in limbal substitutes cellularized with ADSC, BMSC and LESC, a slight signal for WJSC and a very slight positivity for DPSC.

To determine if cells in the cellularized limbal substitutes had a limbal stem cell phenotype, we analyzed the expression of the specific marker Δnp63 in each bioartificial tissue generated by tissue engineering. As expected, expression of this cell marker was highly positive in CTR tissues. In turn, artificial tissues generated with scaffolds decellularized with protocol P6 showed a moderately positive signal only when LESC were used, with a very slight positive signal in BMSC and a negative staining signal in ADSC, DPSC and WJSC. However, the use of scaffolds decellularized with P7 resulted in a slight positive signal for LESC, with the rest of the cellularized tissues being negative for this limbal differentiation marker (Figure 5 and Table 2).

**Figure 6:**
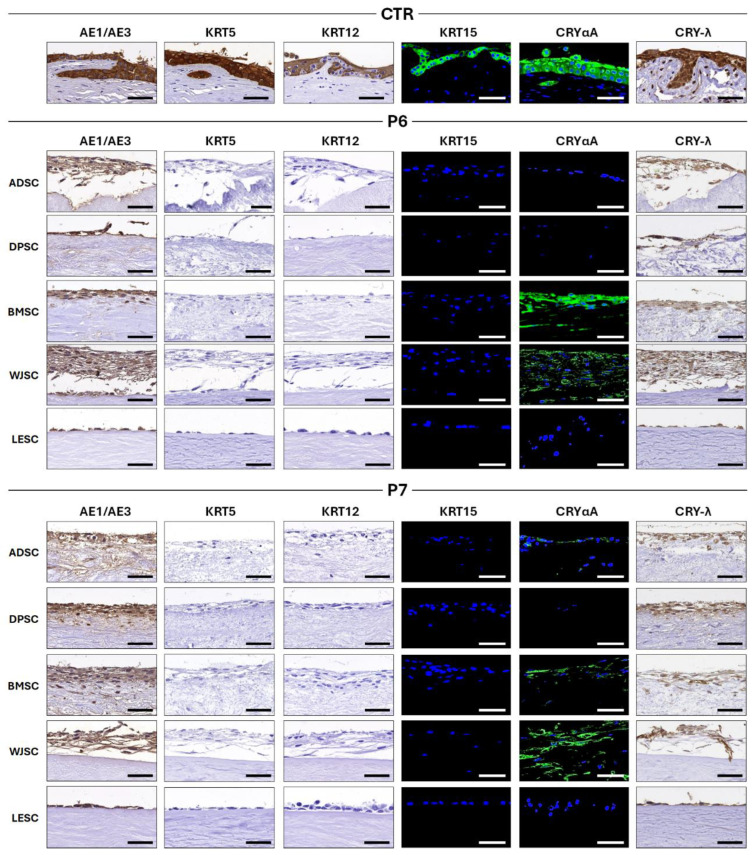
Immunohistochemistry and immunofluorescence analysis of the cellularized human limbal substitutes generated by tissue engineering using decellularization protocols P6 and P7 and control tissues (CTR) for several cytokeratins and corneal crystallins. AE1/AE3: Pancytokeratin AE1/AE3; KRT5: Cytokeratin 5; KRT12: Cytokeratin 12; KRT15: Cytokeratin 15; CRYαA: Crystallin αA; CRY-λ: Crystallin λ. ADSC: Limbal substitutes recellularized with adipose-derived stem cells; DPSC: Limbal substitutes recellularized with dental pulp-derived stem cells; BMSC: Limbal substitutes recellularized with bone marrow-derived stem cells; WJSC: Limbal substitutes recellularized with Wharton’s Jelly-derived stem cells; LESC: Limbal epithelial stem cells. Scale bar = 50 µm.

In addition, cell differentiation was analyzed by determining the immunohistochemical expression of several markers of epithelial differentiation (Figure 6 and Table 2). For pancytokeratin AE1/AE3, CTR native tissues were strongly positive, whereas all bioengineered tissues were moderately positive, regardless of the decellularization protocol and the cell type used to generate these tissue substitutes. Moreover, analysis of cytokeratins KRT5, KRT12 and KRT15 revealed a strong or moderate expression in CTR native tissues, but all bioengineered limbal substitutes resulted to be negative staining for all these markers. For the corneal crystallin CRYαA, our analysis revealed a strongly positive expression in CTR. For scaffolds decellularized with protocol P6, CRYαA expression was strong in tissues cellularized with BMSC, and moderately positive in the WJSC group, but no expression was detected in the ADSC, DPSC and LESC groups. For biomaterials decellularized with protocol P7, the CRYαA signal was moderate when WJSC were used, slight in the BMSC group and very slight when biomaterials were cellularized using ADSC. Finally, the analysis of CRY-λ expression demonstrated that CTR native tissues were strongly positive for this marker, although the staining intensity was moderate in all bioengineered limbal substitutes.

## 4. Discussion

LSCD is a significant ocular disorder affecting an estimated 3.6 individuals per 100,000 population [6], meaning that more than 20,000 persons can be affected by the disease in Europe. Management of these patients imposes a substantial economic burden on healthcare systems, with the expected cost per treatment exceeding USD 7500 [24]. Severe dysfunctions of the human limbus associated with LSCD are important therapeutic challenges, especially when a structural alteration of the limbal niche is present, as is the case in patients with aniridia or deep chemical burns [25]. In these cases, the treatment should be focused not only on restoring a functional population of LESC, but also on restoring the complex three-dimensional structure of the limbal niche, able to maintain the homeostasis of this cell population [16]. For this reason, the development of a fully biomimetic substitute of the human limbus is an unmet clinical need requiring further research. However, the complex structure of the human limbus complicates the development of functional limbal substitutes with potential clinical usefulness, and the number of human limbal substitutes that have been described so far is very low [14,16,17].

In the present study, we have generated a novel human limbus substitute by tissue engineering using several decellularization techniques applied to the human native limbus. The development of decellularization techniques enabled the creation of tissue substitutes reproducing the composition and structure of native tissues in which the donor cells have been removed to prevent immunogenic reactions [14]. Although these methods are promising in the field of tissue engineering of different organs [26], a protocol allowing the generation of a fully biomimetic limbal substitute is yet to be described. In general, decellularization protocols are based on a combination of physical, chemical, and enzymatic methods capable of eliminating all cellular components, without significantly altering the ECM structure and composition, which is critical for the subsequent recellularization process [27]. By applying these protocols, several previous reports described the successful generation of a decellularized limbal substitute using porcine eyes [13,15]. Nevertheless, the histological structure and biochemical composition of tissues may vary among species, and application of a specific protocol may have different effects depending on the origin of the donor organ, thus requiring tissue-specific decellularization optimization [27]. Furthermore, the exact concentration and processing times are crucial factors affecting decellularization efficiency and ECM preservation, and it has been demonstrated that variation in both factors can significantly affect the results of the procedure [28]. Other factors, such as the temperature or the size of the tissue, must be optimized for each tissue type [29].

Since there is no consensus on decellularization protocols applied to the human limbus, we have evaluated seven different protocols based on the main strategies described in the literature. On the one hand, we analyzed the decellularization efficiency of four different protocols (P1 to P4) based on the use of SDS, combined or not with Triton X-100 and nucleases, which we had previously described for porcine cornea decellularization [13]. On the other hand, we analyzed protocol P7, based on the use of SDC and nucleases, resulting in an efficient decellularization, which was used in all previously published protocols applied to the human limbus [14,16,17]. Finally, we evaluated the effects of two novel protocols developed ad hoc in the present work. The first of these protocols (P5) was based on a combination of SB, a zwitterionic detergent that had not been tested on the human limbus, but it demonstrated high efficiency in other tissues, like the peripheral nerve [30], with nucleases. The other protocol (P6) makes use of two different detergents (SB and SDC), combined with nucleases. In P5 and P6, the total time required for limbal decellularization has been reduced, as compared to other protocols. P5 and P6 were formulated with this specific composition based on prior evidence demonstrating the effectiveness of SB-16 (0.6 mM) and SB-10 (125 mM) in other tissue models [30], as well as the high efficiency of SDC and DNase previously shown in P7 [14].

When the decellularizing efficiency of the seven protocols used in the present work was analyzed, we found several differences among the protocols (Figure 1). Although all protocols were capable of decreasing cellular components from the human limbus, we found that protocols P1 to P4, which showed efficacy in porcine limbi, were not able to remove all cells and cell debris from the human limbus, and only protocols P5 to P7 fulfilled the requirements of decellularized tissues for future clinical applications. Interestingly, the combination of several cell-remnant analysis methods, including HE and DAPI staining and DNA quantification, allowed us to determine the decellularization efficiency with high accuracy. Whereas histological staining is able to identify cells and large cell debris, DNA quantification can identify small DNA fragments in a more accurate manner. These results are in agreement with the previous reports related to decellularization of the human limbus, which do not rely on the use of SDS, which is the base of the P1 to P4 protocols used in porcine tissue, but on SDC and nuclease enzymes [14,16,17]. The reasons for this behavior remain unknown, since the porcine cornea is much thicker than the human cornea [31], and one might expect that protocols that demonstrated efficacy in a thick cornea will also be effective in a thin cornea. However, it is well known that the structure and composition of the human cornea stroma differ from the porcine stroma, especially regarding collagen fiber distribution, interfibrillar distance and fibrillar diameter [31,32]. In fact, it has been suggested that tissues in the porcine eye might have a higher degree of disorganization compared to the human eye [33,34], resulting in fewer bonds and a less compact stroma, making decellularization solutions more effective in porcine tissues.

Although further research is needed, we might hypothesize that the shortest interlamellar distance found in the human stroma, which is threefold higher in porcine corneas compared to human corneas [32], could make it difficult for ionic detergents, such as SDS, to access the internal structure of the human tissue. In fact, measurement of interlamellar distance in human and porcine samples using TEM confirmed a 2.8-fold difference between both species (human: 45 ± 5 nm vs. porcine: 125 ± 15 nm, unpublished observations). In the case of SDC, another ionic detergent, it has been demonstrated that its biological effects are different to SDS, and the use of SDC is associated with a higher dissociation of biological molecules treated with this detergent [35], what may explain its increased decellularization efficiency. In addition, zwitterionic detergents, such as SB, have a neutral global electric charge, but contain oppositely charged groups conferring zwitterionic substances high polarity and increased hydrophilicity [36], that may be related to a higher penetrance in the stromal layer of the tissues and, therefore, higher decellularizing efficiency. Furthermore, it has been demonstrated that SB and other zwitterionic detergents may also favor decellularization by activating an apoptotic process in tissues subjected to decellularization [37].

Along with the decellularization efficiency, a proper preservation of the tissue ECM three-dimensional ultrastructure and biochemical composition is an important requirement of tissue decellularization [38]. When the ECM was evaluated in decellularized tissues (Figure 2), we found significant differences among the different decellularization protocols. In line with the decellularization efficiency results, P1 to P4 protocols demonstrated to be associated with an important alteration of the fibrillar and non-fibrillar components of the native limbi, suggesting that these protocols do not fulfill the requirements for human tissue decellularization. Again, the fact that these protocols did show positive results in porcine tissue confirms the intrinsic differences among species and the need to optimize each protocol using human tissue. As for the decellularizing efficiency, the protocols showing the best results in terms of ECM preservation were P5, P6, and P7, which is in agreement with the previous reports using human limbi [14,16,17]. In this milieu, it is well known that SDS is a strong detergent that may cause a significant degradation of ECM components in different types of tissues [27], whilst SB is normally associated with a better preservation of these tissue components [39], probably due to the neutral global electrical charge of zwitterionic detergents. Moreover, the use of SDS is normally associated with protein denaturation of the tissues being decellularized, resulting in a higher level of ECM disruption as compared to nonionic detergents [40]. In contrast, we found that the use of protocols P5, P6 and P7 was not associated with a reduction in the amount of collagen fibers of the decellularized tissue. These results support the use of protocols P5, P6 and P7 to decellularize the human limbus, as an adequate preservation of the fibrillar components of the tissue ECM is crucial for the bioengineered tissue to maintain its biomechanical properties [41]. Regarding non-fibrillar ECM components, we found that the use of SDC was associated with a decrease in proteoglycans and glycosaminoglycans in P6 and P7, whereas proteoglycans were not reduced after decellularization with SB. These results are not unexpected, as the use of zwitterionic detergents, such as SB, is more gentle for the tissue ECM, whereas SDC can affect protein–protein interactions, leading to disruption of the tissue proteoglycans [42].

Another key aspect that must be evaluated in the case of decellularized substitutes is biocompatibility [43], as bioartificial tissue substitutes could be cytotoxic and may induce an immunological reaction in host tissues. In this regard, it has been demonstrated that decellularized tissues may have low biocompatibility to human cells due to the activation of damage-associated molecular patterns [44] that could be related to the presence of cytotoxic agents and rests of decellularizing agents in the decellularized tissue. In this regard, an efficient removal of all remnants of decellularizing agents is crucial for decellularized tissues used clinically, as these agents may compromise cell viability and cell function [28]. Although we did not specifically analyze the presence of remnants of decellularizing agents in the final products, our biocompatibility analyses suggest that these final products were highly biocompatible and were probably free from any remnants of these agents.

To evaluate biocompatibility ex vivo, we used an array of analysis methods based on macrophage cell cultures, as previously reported [45,46] using the three scaffolds showing the most promising results in the previous characterization analysis described above (P5, P6 and P7) (Figure 3). Results confirmed that the use of these scaffolds did not able to impair cell viability, as cell survival was similar in CTR, aCTR, P5, P6 and P7 groups, suggesting that these biomaterials were safe and did not release any harmful molecules to the surrounding environment. An interesting finding was the significant decrease in the cell density of macrophages cultured in the presence of tissues decellularized with P7. Although this phenomenon remains unexplained, cells in contact with these tissues could be experiencing a differentiation process, leading to a proliferative decrease without affecting cell viability, according to the Live/Dead and the TUNEL results. Additionally, we analyzed the pro-inflammatory effects of P5, P6 and P7 biomaterials by determining their capability to induce macrophage polarization. In general, we found that the three types of decellularized biomaterials were associated with a pro-regenerative polarization profile on cultured macrophages, with a CD163/CD86 ratio that was similar or higher to CTR samples and very different from the aCTR group in which a pro-inflammatory profile had been induced. As tissue regeneration and repair are mediated by an adequate equilibrium and immunological balance between M1 and M2 macrophages, in which the CD163/CD86 ratio should be positive, these results are in line with biomaterials intended for use in tissue engineering and confirm that their future in vivo use could be safe for the patient [47,48]. Although a clear threshold has not been established [49], it is well known that the M2/M1 ratio is strongly correlated with the degree of inflammation in diverse types of tissues [49]. In this regard, we previously found that highly biocompatible biomaterials are associated with higher M2 macrophage polarization, with M2/M1 ratios higher than 1 [21], as is the case with the decellularized tissues generated in the present work.

Furthermore, we found that P6 and P7 biomaterials were associated with a release of ROS factors that were similar to CTR, reinforcing the biocompatibility of P6 and P7 biomaterials. However, we found an increase in ROS factors in the P5 group, although dispersion was high and statistical significance was not reached. ROS factors are known mediators of inflammation that can significantly contribute to the development of inflammatory reactions in different conditions [50], and the minimization of ROS factor release should be one of the objectives of tissue engineering. In general, these results confirm the high biocompatibility of tissues decellularized using the P5, P6 and P7 protocols. Although the three groups of samples fulfilled the cell viability, macrophage polarization, and ROS factors release criteria described here, we selected P6 and P7 for further research based on the profile of ROS factors release shown by these biomaterials. Upcoming research should determine whether or not the P5 group of materials could be appropriate for clinical use. In addition, biocompatibility experiments were conducted in the present study using a follow-up time of 24 h based on previous studies demonstrating that this time is sufficient to generate significant effects on macrophage cultures, including cell viability, morphological and metabolic modifications and cell polarization [51,52,53,54]. Future studies should be carried out using longer induction times to determine the long-term effects of these decellularized biomaterials on macrophage cells.

Once the P6 and P7 groups were selected, we used several types of limbal and non-limbal human cells to cellularize both limbal scaffolds. For this purpose, we first used human LESC obtained from the human limbus to reproduce the current gold-standard treatment for patients with limbal deficiency [2]. Results shown in Figure 4 demonstrate that these scaffolds allowed cell viability and cell attachment, and LESC were able to form an epithelial layer in both the P6 and P7 groups, but the typical Vogt palisades were not detected. In this regard, it has been demonstrated that bioengineered tissues generated in the laboratory and kept in culture are partially differentiated, and induction driven by a complex set of endocrine and paracrine signals and factors released by host tissues once the artificial tissue is grafted in vivo is necessary for terminal differentiation [55,56]. In the case of the limbal specialized structures, interesting studies carried out on chicken embryos found that complex interactions between limbal cells and limbal microniche are responsible for generating the palisades of Vogt, and that the limbal epithelium is initially devoid of these structures, which form and mature from a simple epithelial layer [57]. Although additional research is needed, we might hypothesize that in vivo implant of the limbal substitutes may result in the formation of specialized structures, as was the case of the human skin generated by tissue engineering grafted in patients [58].

Then, we generated cellularized limbal substitutes using non-corneal human MSCs obtained from different sources. Although LESC should have the intrinsic potential to generate an efficient and biomimetic limbal substitute, the use of MSC is associated with several potential advantages, including accessibility, high proliferation, differentiation potential and low immunogenicity [22,59], and is considered to be an excellent candidate for allogeneic use [22]. MSC previously showed to have capability to differentiate into corneal epithelial cells, and we were able to generate a biomimetic substitute of the human cornea showing promising results in vivo using MSC [23]. As an ideal source of MSC has not been described, in the present work, we evaluated four types of MSC that demonstrated high potential to differentiate into the epithelial lineage, such as the human skin epidermis [22]. Results showed that all these cell types were able to attach to the decellularized limbal scaffolds with high cell viability, and WJSC and BMSC were able to develop a stratified cell layer more efficiently than LESC, although the Vogt palisades were absent (Figure 4). As previously demonstrated for the human cornea and skin [22,23], these results support the capability of MSCs to generate an epithelial layer potentially useful in tissue engineering and confirm the undifferentiated status of tissues kept ex vivo.

When cell proliferation was analyzed in the different limbal substitutes (Figure 5), we found that most cell types were able to actively proliferate on the surface of the P6 and P7 scaffolds, with WJSC showing the highest proliferation rate of all MSC when cultured on P6 scaffolds. Regarding cell differentiation (Figure 6), we found that, in general, bioartificial tissues tended to show lower expression of typical markers of the human limbus than CTR limbi, confirming again the immature nature of artificial tissues kept in culture. For the limbal stem cell marker Δnp63, our results showed that expression was moderate in P6 scaffolds cellularized with LESC, whereas P7 scaffolds cellularized with the same cell type showed slight positive expression. In turn, the use of P6 scaffolds was associated with a very slight expression of this protein when BMSC were cultured on these biomaterials. In addition, the slight ex vivo expression found in the BMSC group opens the door to the use of this type of non-corneal cells to generate a biological substitute of the human limbus, although additional differentiation induction is needed. Given the important role of Δnp63 in LESC differentiation and function [60], these results could establish the basis for the future in vivo use of these limbal substitutes. When several cytokeratins were evaluated, we found that all bioartificial tissues expressed moderate amounts of pancytokeratin AE1/AE3, a global marker containing several types of cytokeratins, although at lower levels than CTR tissues, although other specific (KRT5, KRT12 and KRT15) were negative in bioartificial tissues. These results confirm that these scaffolds are able to support epithelial differentiation of the cells cultured on their surface, and the epithelial potential of the different types of MSC used in the present work, as previously demonstrated for the skin epidermis [22]. However, the lack of expression of other corneal and limbal markers [61] confirms again that differentiation was only partial and that the in vivo setting may be necessary for terminal differentiation. Although these analyses allowed us to determine cell viability, proliferation, and differentiation of cells cultured on the surface of the novel decellularized limbi, further quantitative analyses carried out at the gene expression level, such as RT-qPCR, and at the protein level, such as ELISA or flow cytometry, should be performed in the future to confirm these results.

In summary, in the present work, we have generated novel models of human limbal substitutes using decellularized scaffolds and different types of cells. Comparison of several decellularization methods allowed us to select some specific protocols able to generate limbal scaffolds fulfilling the requirements for decellularized tissues, including cell removal and ECM preservation, and we found that the use of strong ionic detergents such as SDS is not efficient for the human limbus, and the best results were found when SDC and SB were combined in protocol P6. Although protocols P6 and P7 shared some similarities, the application of P6 resulted in a more efficient differentiation of the cultured cells, suggesting that P6 could be potentially used in future tissue engineering protocols. The fact that protocol P6 requires shorter incubation times than P7 supports the future use of P6 for human limbus decellularization, with subsequent cellularization using corneal or non-corneal cell sources. The efficient preservation of ECM components found in human limbi decellularized with P6 could explain the proper attachment and differentiation levels found by these types of cells cultured on the surface of decellularized scaffolds, as the native scaffold is rich in adhesion motifs promoting cell attachment and cell physiology [62]. Future studies should determine the biomechanical and optical properties of the novel limbal substitutes generated in the present work, as it has been demonstrated that these physical properties are crucial for the proper function of bioartificial tissue substitutes applied to the human eye, especially in the case of the human cornea [42,43].

Although additional clinical studies are needed, we may suggest that the human limbal substitutes generated in this work could be useful for the regenerative treatment of patients with LSCD associated with structural limbal damage. To date, very few tissue-engineered ATMPs have been authorized in Europe or the United States. One of these products is Holoclar^®^, an autologous therapy produced by culturing limbal stem cells on a fibrin-based biomaterial [10]. Although Holoclar^®^ represents an important milestone in tissue engineering [63], its use is restricted to patients with unilateral LSCD who lack structural limbal damage and, therefore, retain an intact limbal niche. In contrast, the product developed in the present work could also benefit patients with limbal structural damage and patients with bilateral LSCD, as the bioartificial limbus could be fabricated using alternative non-limbal MSC sources.

This study has several limitations, including the partial differentiation found in cellularized limbal substitutes, the lack of in vivo experiments carried out in laboratory animals and biomechanical and optical analyses, as well as the reduced sample size in some of the determinations and the lack of a previous estimation of the sample size. In addition, the immunohistochemical analyses carried out on recellularized limbi should be confirmed using complementary quantitative assays. Furthermore, an analysis of potential residues of the decellularizing agents in the final product should be performed before clinical use. If future analysis can demonstrate the potential usefulness of the limbal substitutes generated in the present work, these artificial tissues will need to be adapted to good manufacturing practices fabrication as a novel ATMP fulfilling the requirements established by the National Medicines Agencies for advanced therapies [43].

## 5. Conclusions

In general, the use of the biofabrication methods described in the present work allowed us to obtain human limbal substitutes generated by tissue engineering. On the one hand, we found that protocol P6 could successfully contribute to generating decellularized scaffolds in a short time. On the other hand, recellularization methods allowed the efficient generation of a limbal substitute supporting limbal cell physiology and differentiation that may have future clinical application in patients with structural limbal damage.

## Figures and Tables

**Table 1:** Decellularization protocols evaluated in the present work. P1 to P4 protocols were previously used to decellularize the porcine cornea, P7 previously demonstrated usefulness on the human limbus, and P5 and P6 were developed ad hoc for the present work. The novel protocols developed in the present work have been highlighted in blue.

Reagents	Protocols						
	P1 [13]	P2 [13]	P3 [13]	P4 [13]	P5	P6	P7 [14]
ddH_2_O	24 h	24 h	24 h	24 h	3 × 15 min	3 × 15 min	3 × 15 min
SDS (0.1%)	3 × 24 h	24 h	24 h	24 h	-	-	-
NaCl (1.5 M)	-	2 × 24 h	-	-	-	-	-
ddH_2_O	-	-	3 × 30 min	3 × 30 min	-	-	-
Triton X-100 (0.6%)	-	-	24 h	24 h	-	-	-
ddH_2_O	-	-	3 × 30 min	3 × 30 min	-	-	-
SDC (1%)	-	-	24 h	24 h	-	-	30 min
SB mix solution (SB-16 0.6 mM; SB-10 125 mM)	-	-	-	-	1 h	1 h	-
PBS	-	-	-	-	3 × 30 min	3 × 30 min	3 × 30 min
SDC (0.3%)	-	-	-	-	-	30 min	-
PBS	-	-	-	-	-	3 × 30 min	-
ddH_2_O	-	-	3 × 30 min	3 × 30 min	-	-	-
Trypsin (0.05%)	-	-	-	1 h	-	-	-
Enzymatic solution (DNAse 100 mg/L; RNAse 20 mg/L)	-	-	45 min	45 min	-	-	-
DNAse 1 mg/mL	-	-	-	-	2 h	2 h	O.N. (12 h)
PBS	5 × 15 min	5 × 15 min	5 × 15 min	5 × 15 min	4 × 30 min	4 × 30 min	4 × 30 min
TOTAL PROCESSING TIME (h)	97.25	97.25	102.5	103.5	7.25	9.25	16.75

Reagents, times and concentrations are shown. All detergents were dissolved in double-distilled water. All steps were carried out using slight agitation at room temperature, except for trypsin, DNase and RNase steps, which were incubated at 37 °C. ddH_2_O: double-distilled water; NaCl: Sodium chloride; SDS: Sodium dodecyl sulphate; SDC: Sodium deoxycholate; SB: Sulfobetaine; PBS: Dulbecco’s phosphate-buffered saline, O.N.: Overnight.

**Table 2:** Semiquantitative evaluation of the immunohistochemistry and immunofluorescence analyses performed on cellularized human limbal substitutes generated by tissue engineering using decellularization protocols P6 and P7 and control tissues (CTR).

	PCNA	Δnp63	AE1/AE3	KRT5	KRT12	KRT15	CRYαA	CRYλ
CTR	+++	+++	+++	+++	++	+++	+++	+++
P6-ADSC	++	-	++	-	-	-	-	++
P6-DPSC	+	-	++	-	-	-	-	++
P6-BMSC	±	±	++	-	-	-	+++	++
P6-WJSC	+++	-	++	-	-	-	++	++
P6-LESC	++	++	++	-	-	-	-	++
P7-ADSC	++	-	++	-	-	-	±	++
P7-DPSC	±	-	++	-	-	-	-	++
P7-BMSC	++	-	++	-	-	-	+	++
P7-WJSC	+	-	++	-	-	-	++	++
P7-LESC	++	+	++	-	-	-	-	++

In each sample, cell proliferation was analyzed using the cell proliferation marker PCNA, and epithelial cell differentiation was assessed by determining the cell expression of the limbal marker Δnp63, pancytokeratin AE1/AE3, cytokeratin 5 (KRT5), cytokeratin 12 (KRT12), cytokeratin 15 (KRT15), crystallin αA (CRYαA) and crystallin λ (CRY-λ). The immunohistochemistry and immunofluorescence positive signal were scored as strong (+++), moderate (++), slight (+), very slight (±), or negative (-). ADSC: Limbal substitutes recellularized with human adipose-derived stem cells; DPSC: Limbal substitutes recellularized with dental pulp-derived stem cells; BMSC: Limbal substitutes recellularized with bone marrow-derived stem cells; WJSC: Limbal substitutes recellularized with Wharton’s Jelly-derived stem cells; LESC: Limbal epithelial stem cells.

## Data Availability

The original data generated in the study are openly available at the open access European Research Data Repository Zenodo at https://doi.org/10.5281/zenodo.14867516.

## Supplementary Materials

The following supporting information can be downloaded at: https://www.mdpi.com/article/10.3390/pharmaceutics17121540/s1, Table S1: Technical details used for the immunohistochemical and immunofluorescence analyses carried out in the present work. Table S2: Analysis of decellularized tissue samples using the different protocols described in the present work.

## Abbreviations

The following abbreviations are used in this manuscript:

AB	Alcian Blue
aCTR	Control group of activated macrophages
ADSC	Adipose-derived Stem Cells
ATMP	Advanced Therapy Medicinal Product
BMSC	Bone Marrow-Derived Stem Cells
CRY αA	Crystallin αA
CRY λ	Crystallin λ
CTR	Control
DAPI	4’-6-diamidino-2-phenylindole
ddH_2_O	Double-distilled water
DMEM	Dulbecco’s Modified Eagle’s Medium
DPSC	Dental pulp-derived Stem Cells
ECM	Extracellular matrix
FBS	Fetal Bovine Serum
HE	Hematoxylin-eosin
KRT5	Cytokeratin 5
KRT12	Cytokeratin 12
KRT15	Cytokeratin 15
LESC	Limbal epithelial stem cell
LPS	Lipopolysaccharide
LSCD	Limbal Stem Cell Deficiency
M1	Pro-inflammatory phenotype
M2	Pro-regenerative phenotype
MSC	Mesenchymal Stem Cell
NaCl	Sodium Chloride
O.N.	Over night
P1	Protocol 1
P2	Protocol 2
P3	Protocol 3
P4	Protocol 4
P5	Protocol 5
P6	Protocol 6
P7	Protocol 7
PAS	Periodic acid-Schiff
PBS	Dulbecco’s Phosphate-Buffered Saline
PSR	Picrosirius red
ROS	Reactive oxygen species
SB-10	Sulfobetaine 10
SB-16	Sulfobetaine 16
SDC	Sodium deoxycolate
SDS	Sodium dodecyl sulphate
TUNEL	Terminal deoxynucleotidyl transferase dUTP nick and labeling
WJSC	Wharton’s Jelly-derived Stem Cells
