# Supplementary material for: Generation of a Bioengineered Substitute of the Human Sclero-Corneal Limbus Using a Novel Decellularization Method
Source: Pharmaceutics. 2025 Nov 29;17(12):1540. doi: 10.3390/pharmaceutics17121540 (PMC12736053; doi:10.3390/pharmaceutics17121540)
Supplement: Supplementary file 1 [file pharmaceutics-17-01540-s001.zip › pharmaceutics-3982874-supplementary.pdf]

Antibody/Reagent	Dilution	Pre-treatment	Reference
**Mouse anti-AE1/AE3**	Ready-to-use	EDTA pH=8, 95 ºC for 25 min	Master Diagnostica, Granada, Spain (MAD-001000QD)
**Mouse anti-Δnp63**	1:100	EDTA pH=8, 95 ºC for 25 min	Abcam, Cambridge, UK (Ab172731)
**Mouse anti-crystallin-αA**	1:100	Citrate Buffer pH=6, 95 ºC for 25 min + PBS Tween 20 at R.T for 5 min	Santa Cruz, Texas, USA (SC-28306)
**Rabbit anti-crystallin-λ**	1:50	Citrate Buffer pH=6, 95 ºC for 25 min + PBS Tween 20 at R.T for 5 min	Sigma-Aldrich, Missouri, USA (HPA040403)
**Mouse anti-cytokeratin 5**	Ready-to-use	Citrate buffer pH=6, 95 ºC for 25 min	Master Diagnostica, Granada, Spain (MAD-0000491QD)
**Rabbit anti-cytokeratin 12**	1:250	EDTA pH=8, 95 ºC for 25 min	Abcam, Cambridge, UK (Ab 185627)
**Rabbit anti-cytokeratin 15**	1:75	Citrate buffer pH=6, 95 ºC for 25 min	Abcam, Cambridge, UK (Ab 52816)
**Mouse anti-human PCNA**	Ready-to-use	EDTA pH=8, 95 ºC for 25 min	Master Diagnostica, Granada, Spain (MAD-000903QD)
**DeadEnd Fluorometric TUNEL System**	-	-	Promega, WI, USA (G3250)
**ImmPRESS® HRP Anti-Mouse IgG (Peroxidase)**	Ready-to-use	-	Vector Laboratories, CA, USA (MP-74012)
**ImmPRESS® HRO Anti-Rabbit IgG (Peroxidase)**	Ready-to-use	-	Vector Laboratorioes, CA, USA (MP-74011)
**Anti-mouse IgG FITC antibody**	1:500	-	Sigma-Aldrich, Missouri, USA (F0257)
**Anti-rabbit IgG FITC antibody**	1:500	-	Sigma-Aldrich, Missouri, USA (F9887)
**Cromogen: Diaminobenzidine ready to use kit**	-	-	Vector Laboratorioes, CA, USA (SK-4100)
**Counterstaining: Harris Hematoxylin**	30s	-	Thermo Scientific, Massachusetts, USA (6765004)

SUPPLEMENTARY TABLE S1: Technical details used for the immunohistochemical and immunofluorescence analyses carried out in the present work.

Protocol	Remaining DNA (%)		PSR		AB		PAS	
	Mean	p-value	Mean	p-value	Mean	p-value	Mean	p-value
**CTR**	100±29,17	–	100±4,24	–	100±8,73	–	100±12,95	–
**P1**	86,87±12,53	0,310	83,61±5,68	**<0,001***	62,66±11,12	**<0,001***	53,73±6,02	**<0,001***
**P2**	71,41±14,33	**0,041***	93,09±3,74	**<0,001***	62,23±7,3	**<0,001***	63,65±21,94	**<0,001***
**P3**	34,95±6,78	**0,002***	89,64±5,68	**<0,001***	49,61±9,36	**<0,001***	59,33±8,79	**<0,001***
**P4**	57,72±28,17	0,065	82,29±6,39	**<0,001***	30,39±5,46	**<0,001***	51,51±9,79	**<0,001***
**P5**	3,09±1,23	**0,002***	101,9±4,09	0,378	117,68±5,96	**<0,001***	87,49±9,74	**0,012***
**P6**	2±0,27	**0,002***	105,54±7,25	0,101	62,92±12,7	**<0,001***	61,06±6,44	**<0,001***
**P7**	1,17±0,49	**0,002***	108,02±1,77	**<0,001***	68,33±16,03	**<0,001***	75,29±7,64	**<0,001***

SUPPLEMENTARY TABLE S2: Analysis of decellularized tissue samples using the different protocols described in the present work. Results correspond to the mean and standard deviation of the quantitative values of remaining DNA and picrosirius red (PSR), alcian blue (AB) and PAS staining in each study group (Mean), and to the statistical comparison of each study group with the native human limbus used as a control (CTR) using Mann-Whitney tests. Statistically significant p-values below 0,05 are labeled with asterisks and shown in bold.
